# Comparative Analysis of Volatile and Non-Volatile Metabolites Derived from *Bacillus subtilis* Strains Producing Different Levels of Biogenic Amines

**DOI:** 10.3390/metabo13020219

**Published:** 2023-02-02

**Authors:** Kyuwon Lee, Seo-Hee Kwon, Sumin Song, Do-Yup Lee, Min Kyung Park, Young-Suk Kim

**Affiliations:** 1 Department of Food Science and Biotechnology, Ewha Womans University, Seoul 03760, Republic of Korea; 2 Department of Food and Animal Biotechnology, Seoul National University, Seoul 03760, Republic of Korea; 3 Food Processing Research Group, Korea Food Research Institute, Wanju 55365, Republic of Korea

**Keywords:** biogenic amines, *Bacillus subtilis*, volatile metabolite, non-volatile metabolite

## Abstract

Biogenic amines (BAs), which are mainly generated by the microbial decarboxylation of amino acids, are important nitrogen compounds in fermented foods because of their toxicology. However, amino acids, the precursors of BAs, also play an important role in generating volatile and non-volatile metabolites, which are strongly associated with quality indicators for foods. *Bacillus subtilis* is one of dominant fermentative microorganism in various fermented foods and is well known as a BA-producing bacterium. In this study, *B. subtilis* strains which have different BAs-producing capacities, higher level of BAs production strain (BH) and lower level of BAs production strain (BL), were applied to compare the formations of volatile and non-volatile metabolite profiles according to cultivation times. In this study, histamine, putrescine, and spermidine were detected in all strains, however, 2-phenylethylamine was detected only in BH. Partial least squares discriminant analysis (PLS-DA) was applied to investigate the difference of metabolic profiles according to strains. In BH, some amino acids (phenylalanine, leucine, and threonine) and related volatile metabolites (3-methylbutanoic acid, pyrazines, styrene, and 1H-indole) were produced higher levels. On the other hand, BL produced significantly higher contents of metabolites associated with metabolism of fatty acids and nucleotides. It is necessary to consider the formation of metabolites in terms of quality as well as that of BAs during fermentation.

## 1. Introduction

Biogenic amines (BAs) are nitrogen compounds produced by microbial decarboxylation of amino acids in foods [1]. They can be classified into aliphatic (putrescine, cadaverine, spermine, and spermidine), aromatic (tyramine and phenylethylamine), and heterocyclic (histamine and tryptamine) compounds based on their chemical structure [2]. In general, normal intake of dietary BAs causes no adverse health effects, although, they are considered to be toxic when the capacity of amine-metabolizing enzyme is over-saturated and/or is impaired by specific inhibitors. [3]. Therefore, Food and Drug Administration (FDA) and the European Commission (EC) has set a guidance level for some foods, such as fish and fish sauces. There is individual toxicological threshold, which is few mg/kg, and some BAs, especially histamine, tyramine, and spermine, cause health problems, such as food allergies, skin irritation, headaches, and low blood pressure [4,5,6]. BAs are likely to be present in a variety of foods with high levels of amino acids, including fermented foods, dairy products, fish products, and meat products [7,8]. In particular, it is reported that fermented foods contain considerable contents of BAs because of high contents of amino acids and sufficient decarboxylating microorganisms.

During fermentation, BAs are generated by decarboxylase which catalyze the removal of a carboxyl group of amino acids, before metabolized through acetylation and oxidation by monoamine oxidase (MAO) or diamine oxidase (DAO) [9]. Protease, which is an enzyme to catalyze proteolysis, breaks down proteins and peptides into corresponding amino acids. Since the formation of BAs commonly depends on the presence of amino acids, the activities of proteases as well as decarboxylase and amine oxidase are important for the formation of BAs. On the other hand, further reactions and degradation of BAs into other metabolites also can affect their contents during fermentation. Therefore, the content of BAs and the activity of protease in each microorganism were compared to select higher or lower BAs-producing microorganism.

Previous studies about BAs have mainly focused on determining the contents of BAs in fermented foods. However, amino acids, which act as precursors to BAs, also can convert to some volatile and non-volatile metabolites which are strongly related to desirable or undesirable sensory properties. For example, tryptophan can be converted to 1H-indole, which has an unpleasant odor description (fecal and animal-like odor) [10], as well biogenic amine tryptamine [11]. Therefore, it would be helpful to investigate both BAs and other metabolites derived from amino acids in the same study.

BAs are produced by various microorganisms, including gram-positive bacteria. In this study, *Bacillus subtilis* strains, which showed different BA-producing capacities, were applied to compare volatile and non-volatile metabolic profiles. *B. subtilis* is one of predominant microorganisms in various fermented foods, such as doenjang (a traditional Korean fermented soybean paste) [12]. In preliminary experiment, a total of 31 *B. subtilis* strains, which were isolated from traditional Korean fermented foods, were evaluated on the basis of their total BAs contents and protease activity. In this study, *B. subtilis* strains with a higher or lower ratio of BAs contents than the protease activity was selected. In order to investigate characteristic volatile and non-volatile metabolic patterns, cultured media and cells were collected according to cultivation times and analyzed using gas chromatography-mass spectrometry (GC-MS) and GC-time of flight (TOF)/MS.

## 2. Materials and Methods

### 2.1. Chemicals and Reagents

Tryptone soy broth (TSB) was obtained from Becton Dickinson (Sparks, MD, USA). Acetonitrile and water (HPLC grade) were obtained from J.T. Baker (Phlipsburg, NJ, USA). All the other chemicals were purchased from Sigma-Aldrich (St. Louis, MO, USA).

### 2.2. Strains and Cultivation of Bacillus subtilis

*B. subtilis* strains were supplied by the Korean Collection for Type Cultures (KCTC, Jeongeup-si, Jeollabuk-do, Republic of Korea) and Korean Agricultural Culture Collection (KACC, Jeonju-si, Jeollabuk-do, Korea).

Higher BA-producing strain (BH, *B. subtilis* strain KCTC3014), and lower BA-producing strain (BL, *B. subtilis* strain KACC15938) were selected on the basis of a total content of BAs and protease activity. The contents of the BAs were analyzed with HPLC and protease activity was measured using a skim milk agar. Protease activity was determined by diameter of clear zone after cultivation for 24 h. The BH strain (high BA production) and BL strain (low BA production) were selected based on comparison of the ratio between a total content of BAs and protease activity.

Selected strains were inoculated (initial OD_600_ = 0.1) in 250 mL flasks containing 40 mL of TSB medium after pre-cultivation. Strains were cultivated at 30 °C and 100 rpm for 33 h using a shaking incubator (Vision Scientific Co., Ltd., Daejeon, Chungcheongnam-do, Republic of Korea). The samples were collected according to cultivation benchmarks, e.g., 9 h (exponential time), 17 h (early stationary time), 25 h (stationary time), and 33 h (later stationary time).

### 2.3. Biogenic Amines Analysis

Cultivated samples were separated into cell and medium by centrifugation (4 °C) at 2691× *g* for 30 min. Biogenic amines were extracted from 3 mL of supernatant with 6 mL of perchloric acid (0.4 M in distilled water). 10 µL of 1,7-diaminoheptane (3000 mg/L in distilled water) was applied as an internal standard. The solution was shaken for 30 min using a shaking incubator (Vision Scientific Co., Ltd.,). After shaking, samples were centrifuged at 2691× *g* and 4 °C for 10 min. Saturated NaHCO_3_ solution (300 µL, >8 g/100 mL in distilled water), 200 µL of NaOH solution (2 M in distilled water), and 2 mL of dansyl chloride solution (1 g/100 mL in acetone) were added into 1 mL of the supernatant. The mixture was incubated at 40 °C for 45 min. After incubation, 25% NH_3_OH 100 µL was added and the mixtures were then kept for 30 min at ambient temperature.

The amounts of BAs were quantified using a high-performance liquid chromatograph (HPLC, Agilent Technologies, Santa Clara, CA, USA) equipped with a C_18_ 5 μm column (250 × 4.6 mm, Phenomenex, Torrance, CA, USA) and G1314B variable wavelength detector (VWD) set at 254 nm (Agilent Technologies). The gradient elution system was a mixture of 0.1 M NH_4_CH_3_CO_2_ solution (A) and acetonitrile (B). The gradient procedure started at 50% of solvent A for 15 min, decreased to 10% from 15 min to 20 min, and increased to 50% again from 20 min to 25 min. The injection volume was 20 µL. The HPLC analysis method for BAs was developed from a method used by Yoon et al., (2015) [13]. All experiments were conducted in triplicate.

A series of diluted standard solutions consisting of histamine, putrescine, spermidine, cadaverine, tyramine, and phenylethylamine were used to obtain the calibration curve for each BA. The limit of detection (LOD) and limit of quantification (LOQ) were determined for each.

The equation for LOD is as follows: LOD = 3.3σ/S

The equation for LOQ is as follows: LOQ = 10σ/S

S is the slope of the calibration curves of each biogenic amine, and σ is the relative standard deviation of the y-intercept.

### 2.4. Volatile Metabolites Analysis

The analysis of volatile metabolites was developed from a previous method [14]. Cultivated samples were separated into cell and medium by centrifugation at 2691× *g* and 4 °C for 30 min. The supernatant (8 mL) combined with 2 µL L-borneol (100 mL/L in methanol), as an internal standard, was transferred into a 10 mL glass vial (Agilent Technologies). Stir bar sorptive extraction (SBSE) was applied to extract volatile metabolites using a polydimethylsiloxane-coated stir bar (PDMS twister, 10 nm length, 1.0 mm film thickness, Gerstel GmbH, Muelheim an der Ruhr, Germany) agitated at 1000 rpm for 60 min.

Volatile metabolites were analyzed with a 5977A mass spectrometer (MS, Agilent Technologies) connected to an HP 7890B gas chromatograph (GC) with a Stabilwax column (30 m length, 0.25 mm internal diameter, 0.25 µm film thickness, Resteck, Bellefonte, Pennsylvania, USA). Samples were injected in splitless mode. The temperature of the oven started at 40 °C (5 min) and ramped to 130 °C (5 min) at a rate of 4 °C/min and then ramped up to 220 °C (10 min) at a rate of 4 °C/min. The carrier gas was helium and constant flow rate was 0.8 mL/min. Transfer line temperature was 250 °C, and mass scan range was 35 to 350 m/z.

By comparing the mass spectral data and retention times of metabolites to those of authentic standard compounds, the identification of metabolites was positively confirmed. Otherwise, the retention indexes of metabolites were compared with those from the NIST Chemistry Webbook, and mass spectra data of metabolites were matched with those from NIST08 and Wiley9n.1. Quantitative data were calculated comparing peak areas of compounds to that of the internal standard compound.

### 2.5. Non-Volatile Metabolites Analysis

The non-volatile metabolite analysis was modified from a method used by Yu et al., (2021) [15]. Cultivated samples were separated into cell and medium by centrifugation at 2691× *g* and 4 °C for 30 min. The cells were lyophilized before the extraction of non-volatile metabolites. Lyophilized pallet was dissolved in 1.2 mL of mixed solution (methanol: iso propyl alcohol: water = 3:3:2), and then sonicated for 10 min before being centrifuged at 16,422× *g* and 4 °C for 5 min. The supernatant was transferred into 2 mL tube with internal standards for each metabolite, such as threitol (1000 µg/mL, for sugars and amino acids) and heptadecanoic acid (1000 µg/mL, for organic acids and fatty acids). Then, by using a speed vacuum concentrator (Labogene, Lillerød, Denmark), samples were concentrated to total dryness. The residue was derivatized with methoxyamine hydrochloride in 500 µL of pyridine (40 mg/mL), and then sonicated at 37 °C for 10 min. After sonication, 2 µL of internal retention time index standards, including 13 fatty acids methyl esters (C_8_, C_9_, C_10_, C_12_, C_14_, C_16_, C_18_, C_20_, C_22_, C_24_, C_26_, C_28_, and C_30_), were added before mixture with 45 µL N-methyl-N-trimethylsilyltrifluoroacetamide (MSTFA) containing 1% trimethylsilyl chloride (TMCS, Restek, Bellefonete, PA, USA). Samples were then shaken at 800 rpm and 37 °C for 60 min in a shaking incubator.

Non-volatile metabolites were analyzed with a Leco Pegasus HT time-of-flight mass spectrometer (LECO, St. Joseph, MI, USA) connected to an Agilent 7890B gas chromatograph (Agilent Technologies) with an Rtx-5Sil MS column (30 m length, 0.25 mm internal diameter, 0.25 um film thickness, Restek). Samples were injected in splitless mode and injection volume was 0.5 µL with an Agilent 7693 ALS (Agilent Technologies). The temperature of the oven was started at 50 °C (1 min) and increased to 330 °C (5 min) at a rate of 20 °C/min. The carrier gas was helium and constant flow rate was 1 mL/min. Inlet and transfer line temperatures were 250 °C and 280 °C, respectively. Mass scan range was 85 to 550 m/z at a rate of 20 spectra/sec and EI mode was operated at 70 eV.

Identification of non-volatile metabolites was processed using MS-DIAL. Binbase Library (Fiehn Lab Library, University of California Davis, Davis, CA, USA) was used as a library of standards. The retention index (RI) was determined using fatty acid methyl esters and RI values of each compound were compared with those of standard compounds. By comparing peak areas of metabolites to those of internal standard compounds, non-volatile metabolite quantification was conducted.

### 2.6. Statistical Analysis

Partial least squares discriminant analysis (PLS-DA) was used to determine different metabolites using SIMCA 16 (Umetrics, Umea, Sweden). Additionally, analysis of variance (ANOVA) by Duncan’s multiple range test and t-test were applied to verify the significant differences (*p* < 0.05) between samples using SPSS (version 12.0, Chicago, IL, USA). Heatmap visualization was obtained using a heatmap.2 function in the gplot package implemented in R environment (version 4.2.2).

## 3. Results and Discussion

### 3.1. Quantitative Analysis of Biogenic Amines Production

It is important to set well validated analytical methods to obtain reliable results. Table S1 shows the results of the validation of BA quantitative analysis. The regression coefficients for all BAs were more than 0.99, showing reliability for an optimal setting of quantitative analysis. Limit of detection (LOD) values of BAs, such as 2-phenylethylamine (PHE), putrescine (PUT), cadaverine (CAD), histamine (HIS), tyramine (TYR), and spermidine (SPD), were in the range of 0.03 to 0.15 mg/L, while limit of quantification (LOQ) values were in the range of 0.10 to 0.44 mg/L. A previous study [13] reported that LOD of BAs in liquid food products was in the range of 0.05 to 0.10 mg/L, and LOQ values were in the range of 0.15 to 0.31 mg/L. In this study, LOD and LOQ values covered a similar ranges of previous studies.

Table 1 lists the quantitative results of BAs which were detected in the samples (BL and BH) according to cultivation time. In this study, PHE, PUT, HIS, and SPD were detected in *B. subtilis* samples. There were significant differences between *B. subtilis* strains regarding total BAs contents. A total content of BAs increased along with cultivation times in BH, whereas that decreased in BL overall. In this study, linear relationship between incubation times and the formation of some BAs in BH was not found. The contents of PHE and SPD in BH decreased at 25 h and increased at 33 h. We assumed that the metabolic conversion of BAs to other metabolites was highly occurred at 25 h.

The contents and types of BAs were varied according to *B. subtilis* strain and cultivation times. HIS and SPD were detected in all samples. Similar to the results of total BAs contents, the amounts of HIS and SPD increased according to the cultivation times in BH, whereas those decreased in BL. On the other hand, PUT was found from later cultivation times (>17 h) in all samples, while PHE was detected only in BH. Among polyamines, SPD can be generated from PUT, and agmatine, which is formed from arginine, can be a precursor of PUT [16]. Some BAs serve as precursors for other BAs during fermentation. In the results in BL, the amount of PUT decreased along with cultivation times, whereas that of SPD increased.

### 3.2. Comparison of Metabolic Profiles According to B. subtilis Strains

**Figure 1:**
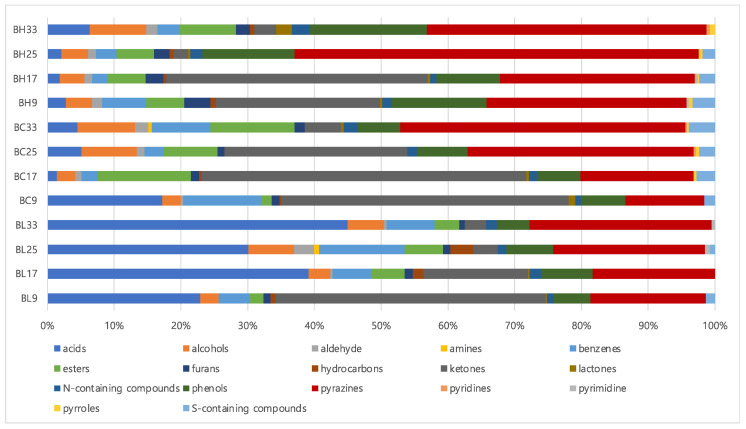
The composition of metabolites in *B. subtilis* samples based on volatile metabolites (BH, high level of BAs- producing strain; BL, low level of BAs-producing strain).

Table S2 lists volatile metabolites identified in *B. subtilis* samples according to cultivation times using SBSE coupled with GC-MS analysis. A total of 68 volatile metabolites were identified: 10 acids, 3 alcohols, 1 aldehyde, 1 amine, 5 benzenes, 5 esters, 2 furans, 4 hydrocarbons, 10 ketones, 5 lactones, 5 phenols, 8 pyrazines, 2 pyridines, 1 pyrimidine, 2 pyrroles, 2 nitrogen-containing compounds, and 2 sulfur-containing compounds. Figure 1 shows the percentages of volatile metabolites contents classified by functional groups. Based on the results, the contents of acids and pyrazines increased with increasing cultivation times, whereas those of ketones decreased in both BH and BL. Among acids, saturated fatty acids, such as hexadecanoic acid and octadecanoic acid, increased at later cultivation time (33 h). In addition, the contents of 2,5-dimethylpyrazine and 2-methylpyrazine increased along with cultivation times. However, the amounts of ketones, especially 3-hydroxybutan-2-one, was suppressed according to the cultivation times. Regarding the composition of volatile metabolites, pyrazines showed the highest proportion in BH, while acids groups constituted almost 24~44% of the total volatile metabolites in BL.

**Figure 2:**
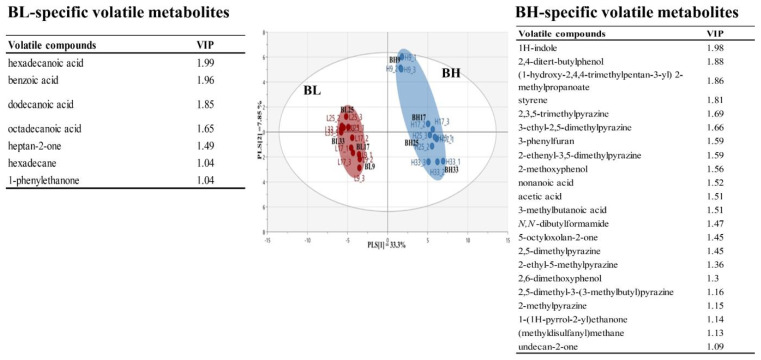
Multivariate statistical model by PLS-DA score plot based on volatile metabolite profiles, and the list of main variables representing BH or BL.

This study applied PLS-DA to investigate differences in the metabolic profiles derived from different *B. subtilis* strains. Figure 2 shows PLS-DA score plot based on volatile metabolite profiles in BL and BH according to cultivation times. The PLS-DA model explained 41.2% of the total variance (PLS [1] and PLS [2] dimensions). The parameters of the cross-validation modeling yielded R^2^Y = 0.990 and Q^2^Y = 0.973. Through the permutation test, values of R^2^ = 0.299 and Q^2^ = −0.361 were obtained. In order to determine the significant metabolic differences between *B. subtilis* strains, a total of 29 main variables contributing to the PLS [1] and PLS [2] dimensions were selected on the basis of a threshold of 1.0 for the variable importance in the projection (VIP). The selected volatile metabolites could been considered as metabolic indicators representing BH or BL. In particular, long-chained fatty acids, including hexadecanoic acid, dodecanoic acid, and octadecanoic acid, were BL-specific metabolites, while, pyrazines, including 2,3,5-trimethylpyrazine, 3-ethyl-2,5-dimethylpyrazine, 2-ethenyl-3,5-dimethylpyrazine, 2,5-dimethylpyrazine, 2-ethyl-5-methylpyrazine, 2,5-dimethyl-3-(3-methylbutyl)pyrazine, and 2-methylpyrazine, were selected as BH-specific metabolites.

**Figure 3:**
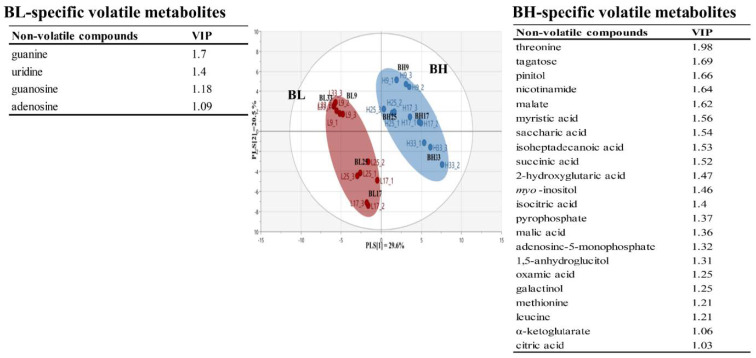
Multivariate statistical model by PLS-DA score plot based on non-volatile metabolite profiles, and the list of main variables representing BH or BL.

Table S3 lists non-volatile metabolites identified in *B. subtilis* samples according to cultivation time using GC-TOF/MS analysis. A total of 68 non-volatile metabolites were identified: 25 amino acids, 5 fatty acids, 14 organic acids, 11 sugars and sugar alcohols, and 12 others. Based on the non-volatile metabolite profiles, Figure 3 shows PLS-DA score plot of BL and BH groups obtained according to cultivation times. The PLS-DA model for BL and BH explained 50.1% of the total variance (PLS [1] and PLS [2] dimensions). The parameters of cross-validation modeling yielded R^2^Y = 0.939, and Q^2^Y = 0.898. The validation parameters (R^2^ = 0.408 and Q^2^ = −0.301) were obtained through a permutation test. A total of 25 significant variables contributing to distinguish between BL and BH groups were selected on the basis of a threshold of 1.0 on the VIP. In the results, amino acids and some metabolites, which were mainly derived from carbohydrate metabolites, were considered as BH-specific metabolites, while some metabolites, which were associated with nucleotides metabolism, were determined to be BL-specific metabolites.

### 3.3. Comparative Analysis of Metabolite Formation Based on Metabolic Pathways

Table S4 shows the changes of amino acids and related BAs according to cultivation times. In this study, there was no clear relationship between the changes in the contents of amino acid precursors and BAs. It might be considered that there are various inter-conversion metabolic pathways between amino acids and BAs.

**Figure 4:**
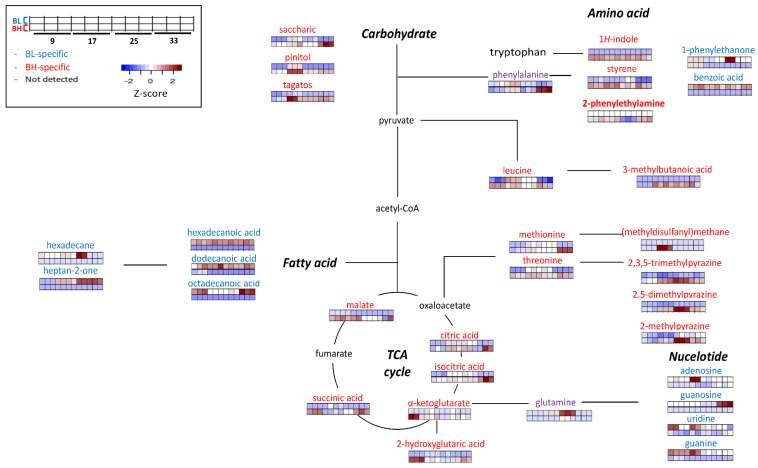
The possible metabolic pathway representing distinctive BH or BL-specificity. Metabolites labeled with red color shows BH-specific metabolites, while blue color represents BL-specific metabolites. The heatmap shows the z-score value of each metabolite, and color intensity represents the magnitude of variations: Red, up-expression; Blue, down-expression.

Figure 4 describes the possible metabolic pathways which were related to BL or BH-specific metabolites. Metabolic features could be indicated mainly in metabolic pathways related with carbohydrates (including TCA cycle), amino acids (especially, phenylalanine, leucine, methionine, and threonine), fatty acids, and nucleotides.

In this study, the metabolic pathways related with amino acids and TCA cycle were significantly more activated in BH than BL. On the other hand, metabolic pathways related with fatty acids, nucleotides and phenylalanine degradation were more activated in BL than BH. In particular, the contents of volatile metabolites derived from aromatic amino acids (including phenylalanine and tryptophan) differed according to each strain. For examples, the content of 1H-indole was higher in BH compared to BL. 1H-Indole, which has an unpleasant odor description (resembling fecal or animal-like odor) [10], is known to be derived from tryptophan or phenylalanine [17]. Styrene, which is a volatile metabolite derived from phenylalanine, was highly formed in BH than in BL. It is one of main volatile metabolites in *Cheonggukjang*, a traditional Korean fermented food [18]. Among BAs, 2-phenylethylamine was detected only in BH. In this study, in BH, BA (2-phenylethylamine) and volatile metabolites (such as 1H-indole and styrene) derived from phenylalanine showed higher amounts compared to BL. It might be explained that phenylalanine-related pathways, including the formation of BAs and certain volatile metabolites (such as styrene and 1H-indole) be highly related to each other.

On the other hand, the content of 1-phenylethanone (acetophenone) and benzoic acid, which are derived from phenylalanine metabolism [19], was higher in BL than BH. In the results, 2-phenylethylamine was not detected in BL, while phenylalanine-derived volatile metabolites, such as 1-phenylethanone and benzoic acid, were detected only in BL. The metabolic pathways related to form these volatile metabolites were more highly activated compared to the pathway of 2-phenylethylamine (BA) in BL fermentation.

Leucine was more significantly generated in BH compared to BL. Also, the contents of volatile metabolite derived from leucine were also higher in BH. 3-Methylbutanoic acid, which is known as an off-odorant in fermented foods, such as traditional Korean fermented soybean paste [20], was found in BH, especially at later cultivation times (>17 h). Since 3-methylbutanoic acid was not detected in BL, it might affect the quality of fermented foods when BL was inoculated as a microbial starter.

Pyrazines were one of the abundant volatile metabolites in BH. Pyrazines mainly has characteristic nutty flavor and contribute to the flavor of fermented soybean paste [21]. The contents of some alkylpyrazines, such as 2,3,5-trimethylpyrazine, 2,5-dimethylpyrazine, 2-methylpyrazine, 3-ethyl-2,5-dimethylpyrazine, 2-ethenyl-3,5-dimethylpyrazine, 2,5-dimethyl-3-(3-methylbutyl)pyrazine, and 2-ethyl-5-methyl pyrazine, were significantly higher in BH. In addition, the contents of threonine, which is known as a precursor of pyrazines [22], was higher in BH compared with BL.

The contents of (methyldisulfanyl)methane, which is a sulfur-containing metabolite with a sulfurous odor description, was higher in BH than BL. Also, the content of methionine, which is a precursor of sulfur-containing metabolites, was significantly higher in BH. In this study, the sulfur-related metabolic pathway might be more activated in BH compared with BL.

Some non-volatile metabolites related with the TCA cycle, such as malate, α-ketoglutarate, 2-hydroxyglutaric acid, citric acid, isocitric acid, and succinic acid, were generated at higher levels in BH compared with BL. In addition, the contents of some sugars and their derivatives, such as tagatose, pinitol, and saccharic acid, were higher in BH compared to BL, regardless of cultivation times.

The contents of saturated fatty acids, such as hexadecanoic acid, dodecanoic acid, and octadecanoic acid, were higher in BL compared with BH. Also hexadecane (related with fatty acids) was generated more significantly in BL. Heptan-2-one, an oxidized metabolite derived from fatty acid, was detected only in BL It has a characteristic blue cheese flavor [23] and is produced by *B. subtilis* during soya bean fermentation [24]. The content of heptan-2-one increased according to cultivation time in BL fermentation.

The contents of some metabolites related to nucleotide degradation metabolism, such as uridine, guanosine, adenosine, and guanine, were higher in BL compared to BH. Nucleotides can be degraded into nucleosides by nucleotidases, that were either further degraded to nucleobases or metabolized into the culture medium [25]. In this study, nucleotide degradation by nucleotidase might be more activated in BL.

## 4. Conclusions

This study compared volatile and non-volatile metabolic profiles and BAs contents of *B. subtilis* strains with different BA-producing capacity. The results demonstrated that the formations of BAs, volatile and non-volatile metabolites were significantly different according to *B. subtilis* strains. In particular, some volatile metabolites related to amino acids (including threonine, leucine and methionine) were generated at higher levels in BH than BL, while those metabolites related with fatty acids and nucleotides were formed more significantly in BL.

Amino acids are not only precursors of BAs, but also those of volatile metabolites. In this study, phenylalanine pathways were highly correlated to the formation of BAs and volatile metabolites. 2-Phenylethylamine (BA) was only found in BH, and the contents of some volatile metabolites derived from phenylalanine (1H-indole and styrene) were higher in BH compared with BH. Based on the results of BH, phenylalanine pathway related to both BAs and volatile metabolites was activated. On the other hand, other volatile metabolites (1-phenylethanone and benzoic acid) derived from phenylalanine could be found in BL.

This study showed that the formation of BAs from amino acids is closely related to those of other metabolites, such as volatiles, related to quality of fermented foods, suggesting that both factors (safety and quality) would be considered for the selection of proper microorganisms during fermentation.

## Figures and Tables

**Table 1:** Quantitative results of biogenic amines (BAs) concentration.

BAs ^a^	Strain ^b^	Results of the Quantitative Analysis of BAs (Mean ± SD) (mg/L)			
		9 h ^c^	17 h	25 h	33 h
PHE	BL	N.D. ^d^	N.D.	N.D.	N.D.
	BH	4.66 ± 0.51 ab	4.70 ± 0.40 ab	3.40 ± 0.06 a	5.12 ± 0.17 b
	*p*-value ^e^	3.97 × 10^−3^	2.41 × 10^−3^	8.73 × 10^−5^	3.89 × 10^−4^
PUT	BL	N.D. a	N.D. a	0.74 ± 0.07 b	0.76 ± 0.05 b
	BH	N.D. a	2.23 ± 0.15 b	2.61 ± 0.10 b	1.60 ± 0.31 ab
	*p*-value	-	1.51 × 10^−3^	1.15 × 10^−5^	3.98 × 10^−2^
CAD	BL	N.D.	N.D.	N.D.	N.D.
	BH	N.D.	N.D.	N.D.	N.D.
	*p*-value	-	-	-	-
HIS	BL	1.71 ± 0.12 c	1.66 ± 0.05 c	1.27 ± 0.05 b	0.96 ± 0.11 a
	BH	1.80 ± 0.11 a	2.38 ± 0.28 ab	1.49 ± 0.08 a	3.08 ± 0.34 b
	*p*-value	3.94 × 10^−1^	4.19 × 10^−2^	1.97 × 10^−2^	4.89 × 10^−3^
TYR	BL	N.D.	N.D.	N.D.	N.D.
	BH	N.D.	N.D.	N.D.	N.D.
	*p*-value	-	-	-	-
SPD	BL	4.68 ± 0.07 b	4.23 ± 0.86 b	5.15 ± 0.14 b	2.31 ± 0.11 a
	BH	5.30 ± 0.38 ab	6.21 ± 0.27 b	3.81 ± 0.16 a	6.24 ± 0.85 b
	*p*-value	1.05 × 10^−1^	1.14 × 10^−2^	4.94 × 10^−4^	1.39 × 10^−2^
Total	BL	6.40 ± 0.15 b	6.31 ± 0.48 b	7.15 ± 0.20 b	4.03 ± 0.09 a
	BH	11.76 ± 0.22 ab	15.52 ± 0.30 bc	11.31 ± 0.32 a	16.04 ± 0.95 c
	*p*-value	4.19 × 10^−6^	9.26 × 10^−6^	4.45 × 10^−5^	2.63 × 10^−5^

Different lowercase letters indicate a significant difference between cultivation times. ^a^ PHE, 2-phenylethylamine; PUT, putrescine; HIS, histamine; SPD, spermidine; CAD, cadaverine; TYR, tyramine. ^b^ BL; *B. subtilis* producing low level of BAs, BH; *B. subtilis* producing high level of Bas. ^c^ Cultivation times. ^d^ Not detected. ^e^ Student *t*-test.

## Data Availability

Data is contained within the article or Supplementary Material.

## Supplementary Materials

The following supporting information can be downloaded at: https://www.mdpi.com/article/10.3390/metabo13020219/s1, Table S1: Results of the validation of biogenic amines (BAs) analysis using HPLC; Table S2: Volatile metabolites identified in *Bacillus subtilis*; Table S3: Non-volatile metabolites identified in *Bacillus subtilis*. Table S4: The changes of amino acid precursors and BAs according to cultivation times.
