# Supplementary material for: Comparative Analysis of Volatile and Non-Volatile Metabolites Derived from Bacillus subtilis Strains Producing Different Levels of Biogenic Amines
Source: Metabolites. 2023 Feb 2;13(2):219. doi: 10.3390/metabo13020219 (PMC9963581; doi:10.3390/metabo13020219)
Supplement: Supplementary file 1 [file metabolites-13-00219-s001.zip › metabolites-2143928-supplementary.pdf]

Comparative Analysis of Volatile and Non-Volatile Metabolites Derived from *Bacillus subtilis* Strains Producing Different Levels of Biogenic Amines

Table S1. Results of the validation of biogenic amines (BAs) analysis using HPLC.

Biogenic amines	Range (mg/L)	Standard curve	Linearity (R^2)	LOD(mg/L)	LOQ(mg/L)
PHE	1-250	y = 0.03x + 0.04	0.9983	0.10	0.31
PUT	1-250	y = 0.14x + 0.06	0.9999	0.15	0.44
HIS	1-250	y = 0.12x + 0.01	0.9999	0.08	0.23
SPD	1-250	y = 0.09x + 0.21	0.9989	0.03	0.10
CAD	1-250	y = 0.11x + 0.26	0.9997	0.53	1.60
TYR	1-250	y = 0.08x + 0.06	0.9998	0.03	0.08

PHE, 2-phenylethylamine; PUT, putrescine; HIS, histamine; SPD, spermidine; CAD, cadaverine; TYR, tyramine.

Table S2. Volatile metabolites identified in *Bacillus subtilis.*

NO. [a]	Volatile metabolites	RI[b]	Relative peak area ratio (Mean±SD) [c]								ID [d]
			BL9	BL17	BL25	BL33	BH9	BH17	BH25	BH33	
Acids											
v16	acetic acid	1461	N.D.[e], a[f]	0.028±0.003b	0.028±0.009b	0.024±0.004b	0.056±0.012ab	0.052±0.009a	0.046±0.022a	0.088±0.022b	A
v24	3-methylbutanoic acid	1670	N.D.a	N.D.a	N.D.a	N.D.a	N.D.a	0.034±0.007b	0.029±0.005b	0.029±0.008b	A
v33	pentanoic acid	1869	N.D.a	N.D.a	N.D.a	0.131±0.039b	N.D.a	N.D.a	N.D.a	N.D.a	A
v42	octanoic acid	2058	N.D.a	N.D.a	N.D.a	N.D.a	N.D.a	N.D.a	N.D.a	0.057±0.002b	A
v45	nonanoic acid	2165	N.D.a	N.D.a	N.D.a	N.D.a	0.055±0.007b	0.028±0.005a	0.026±0.004a	0.104±0.011c	A
v61	dodecanoic acid	2480	0.109±0.045a	0.148±0.035a	0.114±0.005a	0.125±0.020a	0.041±0.007b	N.D.a	N.D.a	N.D.a	A
v65	tetradecanoic acid	>2500	0.256±0.012b	N.D.a	N.D.a	N.D.a	N.D.a	N.D.a	N.D.a	N.D.a	A
v66	(12S)-12-methyltetradecanoic acid	>2500	N.D.a	0.895±0.055b	N.D.a	N.D.a	N.D.a	N.D.a	N.D.a	N.D.a	C
v67	hexadecanoic acid	>2500	2.332±0.081a	2.725±0.104b	2.722±0.231b	2.652±0.214ab	0.105±0.014a	0.194±0.032b	0.133±0.006a	0.329±0.027c	A
v68	octadecanoic acid	>2500	0.684±0.116b	0.276±0.016a	0.325±0.076a	0.889±0.095c	N.D.a	N.D.a	N.D.a	N.D.a	B
Alcohols											
v4	butan-1-ol	1159	0.100±0.009a	0.126±0.004b	0.142±0.018b	0.134±0.002b	0.125±0.028ab	0.093±0.014a	0.145±0.020b	0.132±0.018ab	A
v17	2-ethylhexan-1-ol	1512	0.305±0.021b	0.214±0.044a	0.475±0.028c	0.323±0.022b	0.241±0.027a	0.520±0.033b	0.292±0.031a	0.671±0.049c	A
v64	2-(2-dodecoxyethoxy)ethanol	>2500	N.D.a	N.D.a	0.102±0.100b	N.D.a	N.D.a	N.D.a	N.D.a	N.D.a	C
Aldehydes											
v18	benzaldehyde	1524	N.D.a	0.041±0.016a	0.325±0.265b	0.039±0.006a	0.138±0.012ab	0.199±0.015c	0.130±0.008a	0.155±0.009a	A
Amines											
v63	2-methyl-N-phenylaniline	>2500	N.D.a	N.D.a	0.077±0.029b	N.D.a	N.D.a	N.D.a	N.D.a	N.D.a	C
Benzenes											

v7	styrene	1252	0.048±0.005b	0.042±0.003b	0.056±0.018b	N.D.a	0.122±0.016ab	0.149±0.008b	0.103±0.013a	0.134±0.017b	A
v22	1-phenylethanone	1652	0.367±0.024ab	0.180±0.039a	0.816±0.520b	0.249±0.015a	0.365±0.053b	N.D.a	N.D.a	N.D.a	A
v28	ethyl 2-phenylacetate	1778	N.D.a	N.D.a	N.D.a	N.D.a	N.D.a	0.027±0.002b	N.D.a	N.D.a	B
v35	2-phenylethanol	1931	N.D.a	0.115±0.051b	0.246±0.065c	0.112±0.007b	0.119±0.008a	0.198±0.008b	0.130±0.024a	0.183±0.025b	A
v56	benzoic acid	2427	0.281±0.030a	0.265±0.039a	0.234±0.051a	0.249±0.056a	N.D.a	N.D.a	N.D.a	N.D.a	A
Esters												
v3	3-methylbutyl acetate	1119	N.D.a	N.D.a	0.093±0.058b	N.D.a	N.D.a	N.D.a	0.045±0.005c	0.021±0.004b	A
v12	3-oxobutan-2-yl acetate	1385	0.200±0.027b	0.209±0.091b	0.183±0.017b	0.072±0.016a	N.D.a	0.456±0.016b	N.D.a	N.D.a	B
v34	(1-hydroxy-2,4,4-trimethylpentan-3-yl) 2-methylpropanoate	1893	N.D.a	N.D.a	N.D.a	N.D.a	0.312±0.080a	0.309±0.020a	0.370±0.012a	0.554±0.085b	C
v36	2-phenylethyl 2-methylpropanoate	1954	0.088±0.019a	0.233±0.028b	0.242±0.045b	0.204±0.016b	0.216±0.010c	0.183±0.013b	0.162±0.006a	0.187±0.012b	A
v38	2-phenylethyl 3-methylbutanoate	1978	N.D.a	0.081±0.038b	0.090±0.044b	0.036±0.007ab	N.D.a	N.D.a	0.043±0.013b	0.042±0.012b	A
Furans												
v25	furan-2-ylmethanol	1675	N.D.a	N.D.a	N.D.a	N.D.a	0.022±0.031a	N.D.a	N.D.a	N.D.a	A
v31	3-phenylfuran	1847	0.171±0.003c	0.123±0.015b	0.121±0.010b	0.068±0.005a	0.340±0.030b	0.444±0.046c	0.260±0.020a	0.199±0.037a	B
Hydrocarbons												
v6	dodecane	1200	N.D.a	0.007±0.004b	N.D.a	N.D.a	N.D.a	N.D.a	N.D.a	N.D.a	A
v21	hexadecane	1600	0.044±0.009a	0.061±0.017a	0.182±0.118b	N.D.a	N.D.a	N.D.a	N.D.a	N.D.a	A
v30	octadecane	1800	N.D.a	N.D.a	0.181±0.053b	N.D.a	N.D.a	N.D.a	N.D.a	N.D.a	A
v39	cyclododecane	1994	0.058±0.019b	0.101±0.013c	N.D.a	N.D.a	0.074±0.014a	0.072±0.025a	0.059±0.008a	0.069±0.004a	B
Ketones												
v1	propan-2-one	<1000	N.D.a	N.D.a	N.D.a	0.093±0.006b	N.D.a	N.D.a	N.D.a	N.D.a	C

v5	heptan-2-one	1179	N.D.a	0.020±0.000b	0.026±0.009bc	0.030±0.002c	N.D.a	N.D.a	N.D.a	N.D.a	B
v9	3-hydroxybutan-2-one	1284	5.791±0.383c	1.426±0.154b	0.170±0.025a	0.052±0.003a	2.063±0.192b	6.298±0.302c	0.040±0.012a	0.046±0.011a	A
v20	undecan-2-one	1596	N.D.a	N.D.a	N.D.a	N.D.a	N.D.a	N.D.a	0.014±0.007b	0.013±0.002b	A
v29	1-phenylbutan-1-one	1792	N.D.a	N.D.a	N.D.a	N.D.a	N.D.a	N.D.a	N.D.a	0.033±0.012b	A
v41	pentadecan-2-one	2014	N.D.a	N.D.a	N.D.a	N.D.a	N.D.a	0.026±0.011b	N.D.a	N.D.a	A
v47	1-(2-aminophenyl)ethanone	2213	0.106±0.007a	0.080±0.033a	0.078±0.033a	0.059±0.012a	0.063±0.015ab	0.080±0.012b	0.052±0.010a	0.079±0.004b	A
v48	heptadecan-2-one	2225	N.D.a	N.D.a	N.D.a	N.D.a	N.D.a	N.D.a	N.D.a	0.034±0.003b	A
v58	diphenylmethanone	2462	N.D.a	N.D.a	0.047±0.012b	N.D.a	0.025±0.003b	0.021±0.003ab	0.021±0.002ab	0.017±0.003a	B
v60	2,3-dimethylnaphthalene-1,4-dione	2477	N.D.a	N.D.a	N.D.a	N.D.a	N.D.a	N.D.a	0.015±0.002b	N.D.a	C
Lactones											
v26	2H-furan-5-one	1740	0.021±0.002b	0.028±0.015b	N.D.a	N.D.a	0.035±0.014b	0.026±0.008b	N.D.a	N.D.a	A
v44	5-octyloxolan-2-one	2134	N.D.a	N.D.a	N.D.a	N.D.a	N.D.a	0.057±0.006b	0.037±0.004b	0.061±0.024b	B
v46	6-pentyloxan-2-one	2184	N.D.a	N.D.a	N.D.a	N.D.a	N.D.a	N.D.a	N.D.a	0.012±0.001b	A
v55	6-heptyloxan-2-one	2415	N.D.a	N.D.a	N.D.a	N.D.a	N.D.a	N.D.a	N.D.a	0.108±0.008b	A
v59	6-hexyloxan-2-one	2475	N.D.a	N.D.a	N.D.a	N.D.a	N.D.a	N.D.a	N.D.a	0.045±0.002b	A
N-containing compounds											
v27	N,N-dibutylformamide	1771	N.D.a	N.D.a	N.D.a	N.D.a	N.D.a	0.024±0.004b	0.018±0.008b	0.027±0.006b	B
v43	4-methylquinoline	2110	0.069±0.016a	0.084±0.018a	0.052±0.025a	0.062±0.004a	0.053±0.004a	0.063±0.003b	0.062±0.004b	0.077±0.007c	B
Phenols											
v32	2-methoxyphenol	1856	0.093±0.017a	0.119±0.005b	0.099±0.015ab	0.092±0.002a	0.153±0.015a	0.195±0.010a	0.362±0.022c	0.310±0.046b	B
v40	phenol	2007	0.061±0.020a	0.075±0.028a	0.073±0.005a	0.053±0.013a	0.050±0.014a	0.047±0.019a	0.092±0.027b	0.112±0.015b	A

v51	2,6-dimethoxyphenol	2269	N.D.a	0.141± 0.038b	0.150± 0.060b	0.102± 0.028b	0.100± 0.004a	0.260± 0.044b	0.235± 0.019b	0.285± 0.039b	A
v52	2,4-ditert-butylphenol	2311	N.D.a	N.D.a	N.D.a	N.D.a	0.246± 0.001a	0.358± 0.033b	0.254± 0.014a	0.206± 0.088a	B
v54	4-ethenylphenol	2395	0.264± 0.066c	0.186± 0.051bc	0.151± 0.031b	0.064± 0.003a	0.234± 0.005a	0.236± 0.031a	0.212± 0.017a	0.249± 0.020a	B
Pyrazines											
v8	2-methylpyrazine	1269	0.104± 0.005b	0.079± 0.002a	0.116± 0.019bc	0.125± 0.005c	0.103± 0.010a	0.149± 0.024b	0.232± 0.015c	0.153± 0.036b	A
v10	2,5-dimethylpyrazine	1322	1.624± 0.124b	1.294± 0.045a	1.627± 0.138b	1.661± 0.037b	1.815± 0.096a	2.860± 0.145b	4.432± 0.239c	2.767± 0.161b	A
v13	2-ethyl-5-methylpyrazine	1388	0.195± 0.032b	0.125± 0.022a	0.100± 0.014a	0.085± 0.006a	0.176± 0.016a	0.413± 0.146b	0.384± 0.023b	0.207± 0.013a	B
v14	2,3,5-trimethylpyrazine	1406	0.185± 0.014b	0.132± 0.007a	0.191± 0.019b	0.179± 0.006b	0.248± 0.007a	0.377± 0.025b	0.475± 0.026c	0.347± 0.018b	A
v15	3-ethyl-2,5-dimethylpyrazine	1444	0.167± 0.008c	0.112± 0.010a	0.145± 0.024bc	0.121± 0.009ab	0.215± 0.010a	0.314± 0.032b	0.389± 0.009c	0.240± 0.004a	B
v19	2-ethenyl-3,5-dimethylpyrazine	1541	0.151± 0.015b	0.090± 0.015a	0.115± 0.032ab	0.087± 0.004a	0.146± 0.012a	0.255± 0.017c	0.266± 0.018c	0.200± 0.021b	A
v23	2,5-dimethyl-3-(3-methylbutyl)pyrazine	1655	N.D.a	N.D.a	N.D.a	N.D.a	N.D.a	0.396± 0.028b	0.384± 0.025b	N.D.a	B
v53	2-(2-phenylethyl)pyrazine	2336	0.121± 0.025b	0.077± 0.026ab	0.106± 0.030b	0.057± 0.005a	0.058± 0.007a	0.098± 0.007c	0.102± 0.007c	0.074± 0.004b	B
Pyridines, pyrimidine and pyrroles											
v50	4-pyridin-4-ylpyridine	2242	N.D.a	N.D.a	N.D.a	N.D.a	N.D.a	N.D.a	N.D.a	0.048± 0.016b	A
v62	2,4,6-trimethyl-3-propan-2-ylpyridine	2500	N.D.a	N.D.a	N.D.a	N.D.a	N.D.a	0.030± 0.009b	N.D.a	N.D.a	C
v49	2-phenylpyrimidine	2241	N.D.a	N.D.a	0.083± 0.036c	0.040± 0.001b	0.027± 0.006b	0.038± 0.008c	N.D.a	N.D.a	C
v37	1-(1H-pyrrol-2-yl)ethanone	1973	N.D.a	N.D.a	N.D.a	N.D.a	N.D.a	N.D.a	0.021± 0.005b	0.023± 0.003b	A
v57	1H-indole	2434	N.D.a	N.D.a	N.D.a	N.D.a	0.061± 0.002b	0.052± 0.002ab	0.050± 0.003a	0.048± 0.008a	A
S-containing compounds											
v2	(methyldisulfanyl)methane	1069	0.201± 0.015b	N.D.a	N.D.a	N.D.a	0.266± 0.020b	0.357± 0.018c	0.200± 0.084b	N.D.a	A

v11	(methyltrisulfanyl)methane	1366	N.D.a	N.D.a	N.D.a	N.D.a	N.D.a	0.029± 0.005b	N.D.a	N.D.a	A

[a] Numbered in the order of retention indices (RI).

[b] RI; Retention indices were determined using n-alkanes (C_7– C_{30})

[c] Mean values of relative peak area to the area of internal standard ± standard deviation.

[d] Identification of the compounds was performed as follows: C, mass spectrum was consistent with that of W9N08 and manual interpretation (tentative identification); B, retention index and mass spectrum were consistent with those from the NIST Chemistry Webbook (tentative identification); A, retention index and mass spectrum matched with those of authentic compounds (positive identification)

[e] Not detected.

[f] There are significant differences ($p<0.05$) among each *B. subtilis* sample collected according to cultivation time determined using Duncan's multiple range test between different times having different lowercase letters.

Table S3. Non-volatile metabolites identified in *Bacillus subtilis.*

NO.[a]	Non-volatile metabolites	Qt m/z[b]	Relative peak area ratio (Mean±SD)							
			L9	L17	L25	L33	H9	H17	H25	H33
Amino acids										
A1	ethanolamine	147	0.827± 0.070ab[f]	0.912± 0.072ab	0.710± 0.212a	1.051± 0.024b	0.977± 0.361a	0.911± 0.143a	0.688± 0.039a	1.020± 0.028a
A2	oxoproline	156	1.601± 0.118a	5.564± 0.140c*[e]	5.138± 0.034b*	1.509± 0.069a	3.579± 0.212a*	4.451± 0.024b	4.379± 0.058b	6.129± 0.024c*
A3	α-ketoglutarate	147	0.001± 0.000a	0.006± 0.004a	0.004± 0.005a	0.001± 0.000a	0.032± 0.027a	0.013± 0.008a	0.006± 0.003a	0.009± 0.007a*
A4	citrulline	157	0.004± 0.000a	0.008± 0.003c	0.008± 0.000bc*	0.005± 0.000ab	0.004± 0.001a	0.011± 0.014a	0.006± 0.001a	0.010± 0.001a*
A5	alanine-alanine	116	0.088± 0.007a	1.797± 0.116c*	0.611± 0.517b	0.140± 0.005ab*	0.518± 0.044ab*	0.030± 0.016a	1.133± 0.908b	0.051± 0.016a
A6	tyrosine	218	0.077± 0.008a	0.336± 0.051c	0.228± 0.006b*	0.059± 0.007a	0.062± 0.007a	0.294± 0.040c	0.180± 0.012b	0.380± 0.005d*
A7	glycyl-proline	174	0.001± 0.001a	0.062± 0.021b	0.079± 0.010b*	0.002± 0.000a	0.003± 0.000a	0.035± 0.000b	0.058± 0.007c	0.071± 0.003d*
A8	alanine	116	0.295± 0.047c	0.461± 0.039d*	0.172± 0.006b	0.103± 0.005a	0.473± 0.047c*	0.271± 0.012b	0.167± 0.009a	0.309± 0.021b*
A9	glutamic acid	246	1.816± 0.140b	4.316± 0.169d*	4.065± 0.119c	1.516± 0.033a	2.588± 0.546a	3.833± 0.093bc	3.337± 0.732ab	4.480± 0.318c*
A10	asparagine	100	0.006± 0.001a	0.039± 0.008b*	0.037± 0.002b*	0.010± 0.001a	0.006± 0.001a	0.014± 0.003b	0.012± 0.001b	0.017± 0.002c*
A11	lysine	156	0.358± 0.199a	0.939± 0.041b*	0.793± 0.022b	0.499± 0.012a	0.679± 0.053a	0.710± 0.073a	0.724± 0.046a	0.971± 0.087b*
A12	leucine	299	1.416± 0.156a	2.254± 0.034c	2.002± 0.015b	1.369± 0.133a	2.355± 0.059a*	2.171± 0.048a	2.003± 0.037a	2.089± 0.379a
A13	urea	171	0.050± 0.003b	0.288± 0.037d	0.232± 0.005c*	0.008± 0.005a	0.091± 0.056a	0.268± 0.046c	0.173± 0.011b	0.419± 0.047d*
A14	glycine	174	0.199± 0.015a	0.620± 0.040c	1.272± 0.033d*	0.402± 0.017b	0.496± 0.063a*	0.770± 0.105b	0.708± 0.060b	0.500± 0.019a*
A15	phenylalanine	218	0.168± 0.035b*	0.288± 0.044c	0.155± 0.004b	0.065± 0.005a	0.076± 0.018a	0.242± 0.006b	0.086± 0.067a	0.566± 0.026c*
A16	histidine	154	0.097± 0.012a	0.253± 0.039c*	0.180± 0.010b*	0.074± 0.005a	0.079± 0.004a	0.104± 0.034a	0.099± 0.007a	0.182± 0.002b*
A17	ornithine	142	0.029± 0.004a	0.095± 0.007b	0.101± 0.010b	0.032± 0.001a	0.050± 0.018a	0.061± 0.052a	0.104± 0.002ab	0.128± 0.025b*
A18	cysteine	147	0.043± 0.004a	0.126± 0.073a	0.123± 0.104a	0.051± 0.002a	0.045± 0.021a	0.100± 0.083a	0.038± 0.053a	0.159± 0.086a*
A19	4-hydroxyproline	230	0.003± 0.000a	0.585± 0.015c	0.325± 0.172b	0.004± 0.000a	0.309± 0.028a*	0.312± 0.233a	0.410± 0.025a	0.485± 0.019a

A20	methionine	176	0.098± 0.011b	0.395± 0.026c	0.461± 0.002d	0.060± 0.011a	0.214± 0.030a*	0.583± 0.020b*	0.550± 0.019b*	1.497± 0.017c*
A21	4-aminobutyric acid	174	0.111± 0.019a	0.778± 0.085b	0.972± 0.036c*	0.196± 0.009a*	0.363± 0.044a	0.751± 0.072b	0.722± 0.079b	1.198± 0.127c*
A22	isoleucine	158	0.039± 0.004b*	0.028± 0.007a	0.022± 0.004a*	0.024± 0.003a	0.014± 0.005a	0.057± 0.017b	0.007± 0.001a	0.101± 0.001c*
A23	aspartic acid	232	0.080± 0.068a	0.498± 0.025b	0.594± 0.005c*	0.048± 0.041a	0.436± 0.032b*	0.677± 0.032d*	0.503± 0.013c	0.290± 0.015a*
A24	threonine	117	0.189± 0.024a*	1.081± 0.227b	0.816± 0.593b	0.058± 0.051a	1.828± 0.164ab	2.069± 0.098b*	1.726± 0.018a	2.099± 0.273b*
A25	glutamine	156	0.004± 0.001a	0.306± 0.019b*	1.025± 0.054c	0.038± 0.004a	0.002± 0.000a	0.019± 0.007a	0.165± 0.070b*	0.104± 0.016b*
Fatty acids										
F1	lauric acid	147	0.009± 0.000a	0.025± 0.005a	0.021± 0.018a	0.011± 0.002a	0.014± 0.009a	0.051± 0.013b*	0.051± 0.018b	0.013± 0.014a
F2	myristic acid	117	0.033± 0.002a	0.021± 0.020a	0.027± 0.018a	0.028± 0.001a	0.119± 0.007ab*	0.054± 0.001a	0.064± 0.006a*	0.167± 0.068b
F3	β-hydroxymyristic acid	147	0.015± 0.000a	0.534± 0.453a	0.196± 0.287a	0.020± 0.007a	0.056± 0.003a*	0.048± 0.021a	0.072± 0.070a	0.040± 0.008a*
F4	isoheptadecanoic acid	117	0.077± 0.007a	0.094± 0.026a	0.066± 0.011a	0.082± 0.002a	0.152± 0.001b*	0.108± 0.019a	0.095± 0.018a	0.114± 0.011a*
F5	stearic acid	117	2.156± 0.059a*	2.033± 0.370a	1.978± 0.072a	1.859± 0.040a	1.867± 0.023a	2.070± 0.112a	2.006± 0.028a	2.400± 0.522a
F6	palmitic acid	117	1.578± 0.050ab	1.648± 0.262b	1.476± 0.031ab	1.340± 0.031a	1.577± 0.017a	1.642± 0.020a	1.568± 0.111a	1.651± 0.198a
Organic acids										
O1	oxamic acid	147	0.002± 0.000a	0.455± 0.006c	0.328± 0.016b	0.002± 0.000a	0.589± 0.398ab	0.437± 0.027ab	0.241± 0.011a*	0.871± 0.225b*
O2	maleic acid	147	0.019± 0.002a	0.168± 0.015a	0.189± 0.185a	0.030± 0.002a	0.114± 0.071a	0.274± 0.238a	0.091± 0.061a	1.475± 0.381b*
O3	citraconic acid	147	0.052± 0.004a*	0.135± 0.029c*	0.101± 0.010b	0.025± 0.005a	0.079± 0.011a	0.085± 0.002a	0.091± 0.009a	0.264± 0.061b*
O4	malate	147	0.025± 0.002c	0.016± 0.007b	0.010± 0.002ab	0.005± 0.000a	0.070± 0.014a*	0.064± 0.013a*	0.029± 0.001a*	0.045± 0.042a
O5	threonic acid	147	0.298± 0.003a	0.500± 0.094b	1.333± 0.166c*	0.350± 0.013ab	0.135± 0.078a	0.978± 0.423b	0.333± 0.059a	0.976± 0.271b*
O6	2-hydroxyglutaric acid	129	0.004± 0.000a	0.015± 0.003c	0.008± 0.001b	0.004± 0.000a	0.188± 0.017c*	0.038± 0.004a*	0.023± 0.002a*	0.106± 0.028b*
O7	3-phenyllactic acid	147	0.036± 0.053a	1.025± 0.940a	0.291± 0.049a	0.626± 0.867a	0.189± 0.020a*	0.425± 0.042a	0.108± 0.171a	0.333± 0.266a
O8	3-phosphoglycerate	147	0.012± 0.002a	0.077± 0.010c*	0.054± 0.003b*	0.009± 0.000a	0.066± 0.004b*	0.043± 0.010a	0.033± 0.005a	0.049± 0.014a*

O9	isocitric acid	117	0.010± 0.008a	0.078± 0.003c	0.062± 0.002b	0.017± 0.001a	0.063± 0.007a*	0.096± 0.009a*	0.093± 0.002a*	0.196± 0.053b*
O10	saccharic acid	147	0.002± 0.000a	0.055± 0.016c	0.027± 0.003b	0.002± 0.001a	0.050± 0.006a*	0.089± 0.013b*	0.058± 0.006a*	0.163± 0.028c*
O11	glucosaminic acid	147	0.004± 0.000a	0.010± 0.012a	0.013± 0.015a	0.002± 0.000a	0.007± 0.000a*	0.011± 0.006a	0.070± 0.094a	0.012± 0.014a
O12	lactobionic acid	204	0.012± 0.002b*	0.006± 0.000a	0.004± 0.000a	0.005± 0.000a	0.005± 0.004a	0.015± 0.001b*	0.005± 0.000a	0.012± 0.003b
O13	citric acid	273	0.032± 0.008a	0.226± 0.117b	0.183± 0.085b	0.043± 0.002a	0.106± 0.093a	0.364± 0.039a	0.253± 0.177a	0.450± 0.360a
O14	succinic acid	148	0.052± 0.022a	0.165± 0.261a	0.160± 0.138a	0.016± 0.003a	1.793± 0.130b*	0.400± 0.542a	0.413± 0.307a	1.802± 0.408b*
Sugars and sugar alcohols										
C1	3,6-anhydro-D-hexose	147	0.002± 0.000a	0.071± 0.001ab*	0.143± 0.122b	0.010± 0.001a	0.004± 0.002a	0.011± 0.001a	0.042± 0.015b	0.028± 0.005b*
C2	tagatose	147	0.001± 0.000a	0.003± 0.001c	0.002± 0.000ab	0.002± 0.000bc	0.004± 0.002a	0.023± 0.007c*	0.013± 0.001b*	0.015± 0.002b*
C3	1,5-anhydroglucitol	103	0.003± 0.001ab	0.007± 0.005b	0.001± 0.000a	0.001± 0.000a	0.009± 0.007a	0.016± 0.002a*	0.020± 0.002a*	0.070± 0.018b*
C4	pinitol	147	0.051± 0.005a	0.602± 0.038b	0.070± 0.027a	0.029± 0.001a	0.431± 0.038a*	1.124± 0.044d*	0.623± 0.022b*	0.730± 0.010c*
C5	hexose	147	0.006± 0.002a	0.318± 0.078b	0.069± 0.003a	0.005± 0.001a	0.018± 0.001a*	0.260± 0.036d	0.116± 0.015b*	0.178± 0.004c*
C6	hexitol	147	0.003± 0.000a	0.370± 0.055c*	0.221± 0.022b	0.006± 0.000a	0.011± 0.001a*	0.161± 0.024d	0.070± 0.012b	0.110± 0.008c*
C7	cellobiose	147	0.002± 0.000a	0.004± 0.003a	0.002± 0.000a*	0.003± 0.000a*	0.008± 0.001b*	0.000± 0.000a	0.000± 0.000a	0.001± 0.000a
C8	galactinol	204	0.002± 0.000ab	0.002± 0.001b	0.003± 0.000b	0.001± 0.000a	0.006± 0.000a*	0.011± 0.004b*	0.003± 0.001a	0.002± 0.001a*
C9	fructose	147	0.009± 0.001a*	0.017± 0.007b	0.007± 0.001a	0.007± 0.000a	0.007± 0.000a	0.008± 0.002ab	0.008± 0.002b	0.012± 0.004b
C10	sucrose	147	0.005± 0.005a	0.007± 0.006a	0.028± 0.018b	0.001± 0.001a	0.010± 0.001b	0.002± 0.001a	0.004± 0.003a	0.002± 0.003a
C11	*myo*-inositol	147	0.005± 0.000a	0.042± 0.013c	0.029± 0.001b	0.003± 0.000a	0.013± 0.002a*	0.102± 0.010c*	0.060± 0.008b*	0.117± 0.011c*
Others										
E1	glycerol	147	0.351± 0.004b	0.453± 0.030c	0.374± 0.005b*	0.290± 0.009a	0.292± 0.133a	0.417± 0.019a	0.338± 0.016a	0.360± 0.170a
E2	propane-1,3-diol	147	0.037± 0.063a	2.400± 0.300c	0.667± 0.011b	0.049± 0.006a	0.683± 0.136a*	1.863± 0.201b	0.942± 0.162a	0.571± 0.422a*
E3	pyrophosphate	110	0.016± 0.002a	0.046± 0.013b	0.045± 0.013b	0.014± 0.002a	0.080± 0.019b*	0.053± 0.025ab	0.068± 0.020ab	0.039± 0.004a*

E4	phosphate	299	0.699± 0.137b	0.543± 0.019a	0.518± 0.006a	0.656± 0.059ab*	0.595± 0.072b	0.527± 0.016ab	0.506± 0.006a	0.538± 0.010ab
E5	nicotinamide	179	0.022± 0.001a	0.082± 0.003c	0.052± 0.004b	0.018± 0.001a	0.213± 0.017d*	0.130± 0.004c*	0.072± 0.006a*	0.109± 0.006b*
E6	glycerol-1-phosphate	211	0.630± 0.044a	0.733± 0.132a	0.702± 0.163a	0.649± 0.008a	0.899± 0.020b*	0.542± 0.343ab	0.720± 0.112ab	0.387± 0.335a
E7	glycerol-α-phosphate	299	0.041± 0.007b	0.077± 0.001c	0.089± 0.006d*	0.020± 0.001a	0.091± 0.008a*	0.082± 0.007a	0.061± 0.005a	0.056± 0.049a
E8	guanine	352	0.025± 0.002b*	0.029± 0.007b*	0.008± 0.001a*	0.010± 0.000a*	0.003± 0.000c	0.001± 0.000b	0.001± 0.000a	0.002± 0.000c
E9	uridine	147	0.018± 0.007b*	0.018± 0.007b*	0.012± 0.001ab*	0.006± 0.004a	0.005± 0.002a	0.003± 0.003a	0.006± 0.001a	0.008± 0.004a
E10	adenosine	169	0.004± 0.001a*	0.010± 0.006b	0.003± 0.000a*	0.004± 0.000a	0.002± 0.000a	0.002± 0.000a	0.002± 0.000a	0.003± 0.001b
E11	guanosine	147	0.023± 0.002b*	0.017± 0.003ab*	0.004± 0.000a*	0.111± 0.014c*	0.001± 0.000a	0.003± 0.000c	0.001± 0.000a	0.002± 0.000b
E12	adenosine-5-monophosphate	169	0.053± 0.010a	0.189± 0.047b	0.166± 0.020b*	0.035± 0.000a	0.291± 0.033b*	0.190± 0.120ab	0.128± 0.010a	0.293± 0.028b*

[a] Numbered in the order of retention indices (RI).

[b] Quantitative m/z

[c] Mean values of relative peak area to the area of internal standard ± standard deviation.

[d] The meaning of the letters and numbers inside the score plot is the following: BL9, 17, 25 and 33 (lower level of BAs producing *B. subtilis* strain obtained at 9, 17, 25 and 33 h in replication, respectively), BH9, 17, 25 and 33 (higher level of BAs producing *B. subtilis* strain obtained at 9, 17, 25 and 33 h in replication, respectively)

[e] There are significant differences ($p<0.05$) among samples collected according to strains determined using t-test between different samples having '*'.

[f] There are significant differences ($p<0.05$) among samples collected according to cultivation time determined using Duncan's multiple range test between different times having different lowercase letters.

Table S4. The changes of amino acid precursors and BAs according to cultivation times.

Metabolite	Sample	Cultivation time			
		9h	17h	25h	33h
Histidine	BL[a]	1.475[b]	-0.285	-0.465	-0.725
	BH	-0.817	-0.265	-0.375	1.457
Histamine	BL	0.878	0.736	-0.368	-1.246
	BH	-0.554	0.275	-0.996	1.275
Phenylalanine	BL	-0.011	1.299	-0.153	-1.135
	BH	-0.728	-0.002	-0.684	1.415
2-Phenylethylamine	BL	-[c]	-	-	-
	BH	0.256	0.310	-1.440	0.875
Ornithine	BL	-0.902	0.787	0.941	-0.825
	BH	-0.978	-0.677	0.499	1.156
Glutamine	BL	-0.715	-0.079	1.438	-0.644
	BH	-0.926	-0.703	1.215	0.414
Putresine	BL	-0.866	-0.866	0.843	0.889
	BH	-1.398	0.539	0.869	-0.009
Spermidine	BL	0.471	0.110	0.849	-1.430
	BH	-0.079	0.719	-1.386	0.746

[a] The meaning of the letters is the following: BL (lower level of BAs producing *B. subtilis* strain), BH (higher level of BAs producing *B. subtilis* strain).

[b] z-score vlaues

[c] Not detected
